# Electroacupuncture Reduces Anxiety Associated With Inflammatory Bowel Disease By Acting on Cannabinoid CB1 Receptors in the Ventral Hippocampus in Mice

**DOI:** 10.3389/fphar.2022.919553

**Published:** 2022-07-08

**Authors:** Xue-Fei Hu, Hong Zhang, Ling-Ling Yu, Wen-Qiang Ge, Ou-Yang Zhan-mu, Yan-Zhen Li, Chao Chen, Teng-Fei Hou, Hong-Chun Xiang, Yuan-Heng Li, Yang-Shuai Su, Xiang-Hong Jing, Jie Cao, Hui-Lin Pan, Wei He, Man Li

**Affiliations:** ^1^ Department of Neurobiology, School of Basic Medicine, Tongji Medical College of Huazhong University of Science and Technology, Wuhan, China; ^2^ Institute of Integrated Traditional Chinese and Western Medicine, Tongji Hospital, Tongji Medical College, Huazhong University of Science and Technology, Wuhan, China; ^3^ Institute of Acupuncture and Moxibustion, China Academy of Chinese Medical Sciences (CACMS), Beijing, China; ^4^ Department of Neurology, Tongji Hospital, Tongji Medical College, Huazhong University of Science and Technology, Wuhan, China; ^5^ Department of Anesthesiology and Perioperative Medicine, The University of Texas MD Anderson Cancer Center, Houston, TX, United States

**Keywords:** acupuncture, visceral pain, anxiety, CB1 receptors, ventral hippocampus

## Abstract

The therapeutic effects of electroacupuncture (EA) on the comorbidity of visceral pain and anxiety in patients with inflammatory bowel disease (IBD) is well known. It has been known that the ventral hippocampus (vHPC) and the cannabinoid type 1 receptors (CB1R) are involved in regulating anxiety and pain. Therefore, in this study, we determined whether EA reduces visceral pain and IBD-induced anxiety *via* CB1R in the vHPC. We found that EA alleviated visceral hyperalgesia and anxiety in TNBS-treated IBD mice. EA reversed over-expression of CB1R in IBD mice and decreased the percentage of CB1R-expressed GABAergic neurons in the vHPC. Ablating CB1R of GABAergic neurons in the vHPC alleviated anxiety in TNBS-treated mice and mimicked the anxiolytic effect of EA. While ablating CB1R in glutamatergic neurons in the vHPC induced severe anxiety in wild type mice and inhibited the anxiolytic effect of EA. However, ablating CB1R in either GABAergic or glutamatergic neurons in the vHPC did not alter visceral pain. In conclusion, we found CB1R in both GABAergic neurons and glutamatergic neurons are involved in the inhibitory effect of EA on anxiety but not visceral pain in IBD mice. EA may exert anxiolytic effect *via* downregulating CB1R in GABAergic neurons and activating CB1R in glutamatergic neurons in the vHPC, thus reducing the release of glutamate and inhibiting the anxiety circuit related to vHPC. Thus, our study provides new information about the cellular and molecular mechanisms of the therapeutic effect of EA on anxiety induced by IBD.

## 1 Introduction

Inflammatory bowel disease (IBD), including Crohn’s disease and ulcerative colitis, is a prevalent clinical problem worldwide (Mittermaier et al., 2004; Molodecky et al., 2012; Neuendorf et al., 2016). In addition to gastrointestinal symptoms, patients with IBD also experience emotion disorders, such as anxiety and depression (Ng et al., 2017), which in turn exaggerate gastrointestinal symptoms (Ananthakrishnan, 2015). Several reports have shown that the comorbidity of visceral pain and anxiety in IBD mice resulted from the bidirectional communication between the gut microbiota and the brain (Moloney et al., 2016; Banfi et al., 2021). Electroacupuncture (EA) is an effective treatment for gastrointestinal diseases, pain symptoms and mood disorders through electrical stimulation of needles inserted into specific acupoints (Wu et al., 1999; Errington-Evans, 2012; Lv et al., 2019; Smith et al., 2019; Song et al., 2019; Wang et al., 2019). However, the underlying mechanism of the therapeutic effects of EA on IBD remains largely unknown.

Several pieces of evidence indicate that the ventral hippocampus (vHPC) plays a key role in modulating anxiety-like behaviors (Allsop et al., 2014; Bannerman et al., 2014; Shah et al., 2021) and pain process (Fasick et al., 2015; Vasic and Schmidt, 2017; Liu et al., 2018). Pathological anxiety and chronic stress lead to structural degeneration and impaired function of the hippocampus (Mah et al., 2016; Price and Duman, 2020). In addition, the hippocampus plays an important role in the development and maintenance of pain disorder (Fasick et al., 2015; Vasic and Schmidt, 2017). It needs to be determined whether EA attenuates IBD induced visceral pain and anxiety *via* the vHPC.

Cannabinoid type 1 receptors (CB1R) are highly expressed in the brain areas (Evans and Van'T, 1975; Tsou et al., 1998; Fletcher-Jones et al., 2020) and are related to the control of anxiety response (Haller et al., 2004; Rey et al., 2012) and pain process (Padilla-Coreano et al., 2016; Wang et al., 2020), such as vHPC in particular. CB1R are mainly located at presynaptic terminals and inhibit the release of several classic neurotransmitters, including glutamate and GABA (Egertova et al., 1998; Lisboa et al., 2015). CB1R are preferentially expressed in GABAergic neurons (Hill et al., 2013), but less expressed in glutamatergic neurons (Katona and Freund, 2012). Moreover, CB1R in glutamatergic and GABAergic neurons in the cortex have an opposing role in controlling anxiety-like behaviors (Lafenetre et al., 2009; Haring et al., 2011; Ruehle et al., 2013). However, little is known about the role of CB1R in glutamatergic and GABAergic neurons in the vHPC in visceral pain and anxiety in IBD. It is also unclear whether EA attenuates these symptoms via CB1R expressed in the vHPC.

Based on these pieces of evidence, the present study investigated whether EA alleviate visceral pain and anxiety in 2,4,6-trinitrobenzene sulfonic acid (TNBS)-induced IBD mice. Then, we observed the distribution of CB1R in glutamatergic or GABAergic neurons in the vHPC before and after TNBS and EA treatment. We determined whether genetically ablating CB1R expressed in glutamatergic or GABAergic neurons in the vHPC altered the effects of EA on visceral pain and anxiety in IBD mice. Our findings provide new evidence that CB1R expressed in the vHPC might be involved in the effects of EA on IBD induced anxiety, but not on IBD induced visceral pain.

## 2 Materials and Method

### 2.1 Animals

Adult male C57BL/6 mice (8 weeks old; 20—25 g) were raised in home-cages in the environment of 23°C ± 2°C and a 12 h light/dark cycle. The mice had free access to food and water. All CB1R-flox mice (mCnr1flox/flox) and their wild-type littermate (WT) mice (male, aged 8 weeks, and 18–21 g) were bought from the Cyagen biosciences laboratory (Nanjing, China). All animal procedures were approved by the Institutional Animal Care and Use Committee at Huazhong University of Science and Technology and conformed to the ethical guidelines of the International Association for the Study of Pain and Anxiety.

### 2.2 Viruses Constructs and Surgery

The Cre/loxP system (Kos, 2004) was used to delete CB1R in GABAergic and glutamatergic neurons. The recombinant adeno-associated viruses (rAAV)-mDIX-cre-WPRE-pA viruses were used to delete CB1R in GABAergic neurons. The rAAV-CaMKII-cre-WPRE-pA viruses were used to delete CB1R in glutamatergic neurons. All viruses used in this research were purchased from the Brain VTA scientific and technical corporation (Wuhan, China).

Before microinjection, the mice received an intraperitoneal injection (i.p.) of 100 mg/kg of tribromoethanol for anesthesia and were fixed in the stereotaxic apparatus (RWD Instruments, China). An incision with a length of 1.5 cm was made along the midline of the skull and the periosteum on the surface of the skull was removed. Then, a small hole was grinded by ironic rotor. Viruses injection was performed based on the coordinate of vHPC (2.95 mm backward from the bregma, 2.75 mm lateral from the midline, and 3.75 mm ventral to the skull) (Allsop et al., 2014). Designed viruses vectors (200 nl) were injected into vHPC at a rate of 50 nl per 60 s. Data were excluded from analysis if the viruses infection exceeded area of the vHPC.

### 2.3 IBD Model

IBD was induced in mice as described previously (Silva et al., 2019). Mice were anesthetized with tribromoethanol. A PVC-Fr4 catheter (*Φ* 2.7 mm, YN Medical Instrument, Yangzhou, China) lubricated by corn oil was inserted into the anus to the colon at a distance of 4 cm, and the other end of PVC-Fr4 catheter was attached with a 1 ml syringe. The TNBS intra-rectal (IR) solution included 50 μl of 5% w/v TNBS solution (Sigma-Aldrich, St. Louis, MO, United States) and 50 μl absolute ethanol, which was injected into the colon of anesthetized mice. Mice of the vehicle control group received an injection solution comprised of 50 μl distilled water and 50 μl absolute ethanol. After injection, mice were kept in an upside-down position for 5 min to prevent solution leakage. The mice were placed on a heating pad until recovery from anesthesia.

### 2.4 EA Treatment

EA treatment was applied to bilateral “Dachangshu” (BL25) acupoints 1 day after TNBS injection. The BL25 acupoints were located at both side of the waist and 7 mm lateral to the fourth lumbar spinous. After mice were restrained by specialized fabric equipment, acupuncture needles were inserted into acupoints with a depth of 2.5 mm. Then, acupuncture needles were connected to an EA stimulator (Huatuo brand, Suzhou, China) and electrical stimulation pulses (1 mA, 2 Hz, intermittent wave) were applied for 30 min.

As for sham EA, acupuncture needles only adhered to the specific points, neither penetrated the skin nor received electrical stimulation pulses. Half an hour after EA treatment, the related behavior tests were performed.

### 2.5 Behavioral Tests

#### 2.5.1 Measurement of Visceral Hyperalgesia

Measurements were made continuous 5 days after TNBS injection. The colorectal distension (CRD) method was used to obtain the abdominal withdrawal reflex (AWR) score of mice to evaluate the degree of visceral hyperalgesia. The mice were placed in a plexiglass compartment (20 cm × 20 cm × 10 cm) for 5 min. The PVC-Fr4 catheter was inserted into the latex balloon (length 4–5 cm), and the end of the balloon was firmly tied to the test catheter. The catheter is connected with one end of the disposable medical three-way tubing, one end of the three-way tubing was connected to a sphygmomanometer. The other end is connected with 20 ml disposable syringe, and 20 ml of 20°C water was injected into the syringe. The balloon was inserted into the rectum until the catheter reached the colon (2 cm from the end of the balloon). The balloon catheter was fixed to the bottom of the tail to prevent it from sliding out.

The CRD test was performed in a step-by-step compression mode (20/40/60/80 mmHg). Each pressure value was measured twice. Each test lasted 30s with an interval of 4 min. The AWR score was calculated based on Al-Chaer’s method (Al-Chaer et al., 2000): no behavioral response to CRD was rated as 0 point, short pauses in head or body movements during stimulation was rated as 1 point; abdominal muscle contraction during stimulation was rated as 2 point; abdominal lifting was rated as 3 point; body arch, pelvic cavity or scrotum lifting was rated as 4 point.

#### 2.5.2 Open Field Test

OFT was used to evaluate anxiety-related behaviors of mice (Choleris et al., 2001). Mice were placed in the center of a polystyrene enclosure (50 cm × 50 cm × 50 cm) and recorded by videotape instrument for 5 min. The center area was defined as the centric 25 cm × 25 cm area. The open field was cleaned with 75% ethanol between each trial and the track was analyzed using LabState software (Xinruan information technology co., LTD, Shanghai, China). Time spent in the center area was recorded.

#### 2.5.3 Elevated Plus-Maze

Anxiety-related behaviors were also tested on an EPM apparatus, which was comprised of 100 cm open arms and 100 cm close arms (Campos et al., 2013). Free-moving mice were recorded by videotape instrument for 5 min. The elevated plus-maze was cleaned with 75% ethanol between each trial and the track was analyzed using ANY-maze software (Xinruan information technology co., LTD, Shanghai, China). Time spent in the open arm was recorded.

### 2.6 Immunofluorescence Labeling

Mice were deeply anesthetized with tribromoethanol and were transcardially perfused with 100 ml of 37°C normal saline followed by 50 ml of 4% paraformaldehyde in 0.1 M phosphate buffer (PBS, pH 7.4) at 4°C for fixation. The brain tissues were quickly separated and post-fixed for 6–8 h in the same fixative solution and dehydrated in 20% sucrose in 0.1 M PBS for 24 h and 30% sucrose in 0.1 M PBS for 24 h at 4 C. The brain were removed immediately and post-fixed in PFA. The optimal cutting temperature compound (OCT) embedded blocks were sectioned to 30 μm thickness.

Sections from each group were rinsed in 0.01M PBS and blocked for 2 h with blocking solution (5% donkey serum and 0.2% Tween 20 in 0.01 M PBS) at room temperature. The sections were incubated with the following antibodies: rabbit anti-CB1R (1:500, Santa Cruz, United States), mouse anti-neurogranin (1:1000, Abcam, United States), guinea pig anti-GABA (1:1000, Abcam, United States) and mouse anti-GAD 67 (1:500, Abcam, United States). Subsequently, the free-floating sections were washed with 0.01M PBS 3 times and incubated with following secondary antibodies for 2 h: donkey anti-rabbit IgG conjugated with Dylight 594 (1:500, Abcam, United States), donkey anti-rabbit IgG conjugated with Dylight 488 (1:500, Abcam, United States), donkey anti-mouse IgG conjugated with Dylight 594 (1:500, Abcam, United States), goat anti-mouse IgG conjugated with Dylight 488 (1:500, Abcam, United States), and goat anti-guinea pig IgG conjugated with Dylight 594 (1:500, Abcam, United States). The sections were washed 3 times in 0.01M PBS and then cover-slipped. Olympus BX51 fluorescence microscope was used to view the sections, and images were captured using Qimaging Camera and QCapture software. Images were analyzed using the NIH Image J software (Bethesda, MD, United States). The layouts of the images were based on Photoshop CS5 (ADOBE Company, United States).

### 2.7 Western Blotting

Mice were deeply anesthetized with tribromoethanol and were transcardially perfused with 100 ml of 37°C normal saline. The brain tissues (vHPC regions) were immediately removed and stored at -80°C. The tissues were lysed by adding 40 mg/ml RIPA lysis buffer (Biosharp, China) and 40 mg/ml phenylmethyl sulfonyl fluoride (Biosharp, China) to the samples for 30 min. The lysed tissues were centrifuged at 12,000 rpm for 15 min at 4°C and supernatant liquids were collected. The protein contents were quantified by using the Enhanced BCA Protein Assay Kit (Beyotime Biotechnology, China).

The protein (40 mg) was denatured in loading buffer at 95°C for 5 min, separated on a 10%/12% glycine-SDS-PAGE gel (Beyotime Biotechnology, China), and then transferred onto a PVDF membrane (Millipore Immobilon-P, United States). The membranes were blocked with 5% BSA (Beyotime Biotechnology, China) at room temperature for 1 h, followed by incubation with primary antibodies at 4°C overnight: rabbit anti-CB1R antibody (1:500, Santa Cruz, United States) and rabbit anti-GAPDH antibody (1:1000, Thermo Scientific, United States). The membranes were washed in 0.01M Tris-HCI buffer salt solution and 0.2% Tween 20 (TBST) 6 times and incubated with following secondary antibodies for 2h: goat anti-rabbit IgG (1:5000, Abcam, United States). The signals were recorded using Super Signal West Pico chemiluminescent substrate (Thermo Scientific, United States). The densitometric analysis of the protein band images was performed using the NIH Image J software (Bethesda, MD, United States).

### 2.8 Statistical Analysis

The analysis for behavioral tests was performed by experimenters who were blinded to the treatment. All data were presented as mean ± standard errors of means (s.e.m.), unless otherwise specified. Each data set was firstly tested for normal distribution and those fitted Gaussian distribution were used for parametric analysis. Student *t*-test (paired or unpaired) was used for comparison between two groups and one-way analysis of variance was used to analyze the difference among more than two groups, followed by Tukey post-hoc comparison. When two independent variables were considered, two-way ANOVA was used. For those data that did not fit the Gaussian distribution, Wilcoxon matched-pairs rank test was used for paired comparison and Kolmogorov-Smirnov test was employed to compare between two independent samples. A statistical significance was defined as *p <* 0.05. All statistical analysis and data plotting were performed by GraphPad Prism ver8.0 (GraphPad Inc, United States).

## 3 Results

### 3.1 EA Alleviated Visceral Hyperalgesia and Anxiety in TNBS-Treated IBD Mice

**FIGURE 1:**
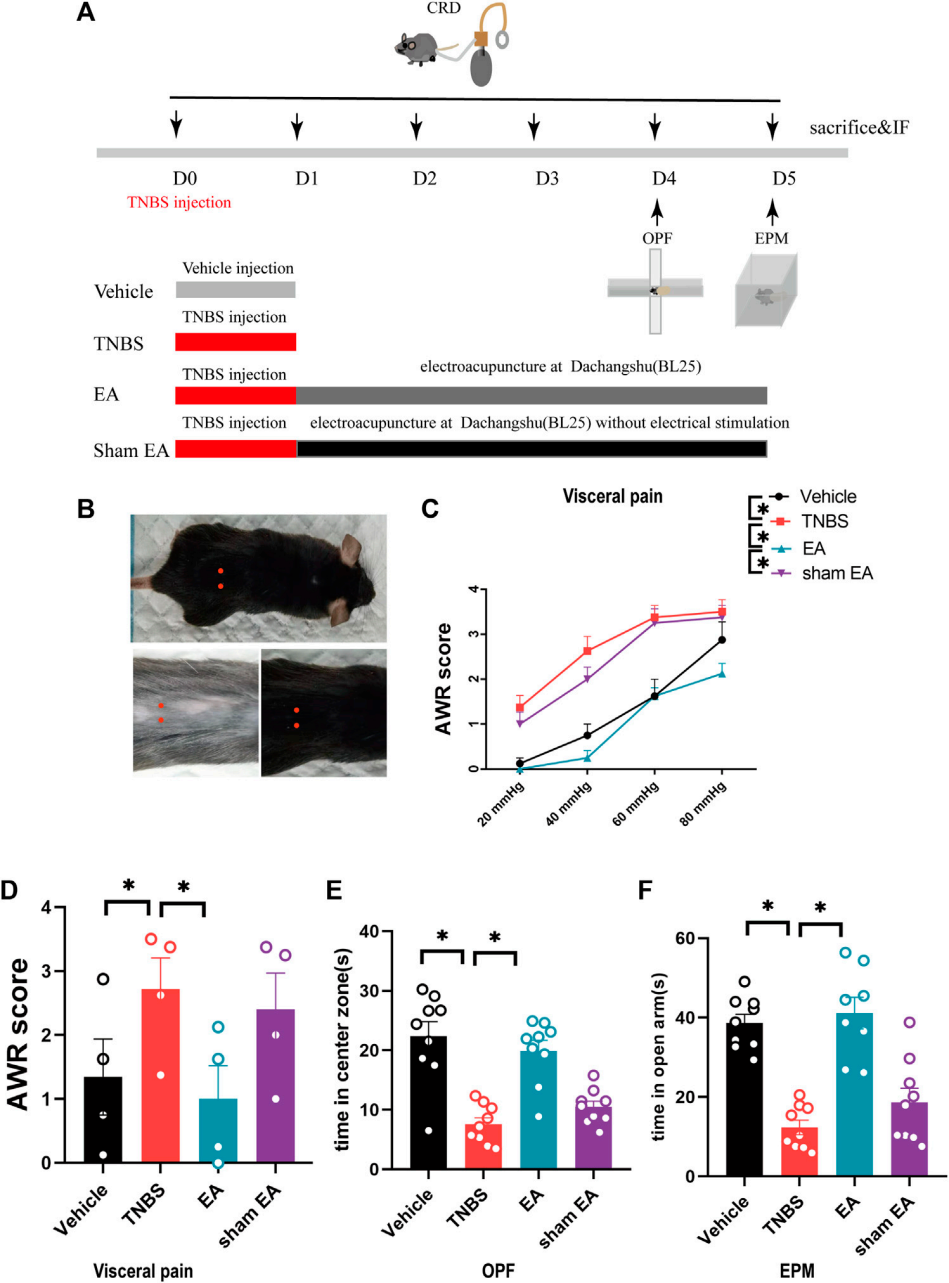
EA relieved the visceral hyperalgesia and anxiety-like behaviors of IBD mice. **(A)** Experimental flowchart. **(B)** Schematic diagram of Dachangshu points (BL25) on the skin surface. **(C,D)** Visceral hyperalgesia was evaluated by CRD. **(E)** Anxiety-related behaviors were recorded as time in center zone in the OPF. **(F)** Anxiety-related behaviors were recorded as time in open arms in the EPM. The data are expressed as mean ± SEM (*n* = 9 mice). *represents *p* < 0.05 between marked groups.

Five days after TNBS injection, the AWR score of CRD in TNBS group was significantly increased compared with vehicle control mice (Figures 1A–D, *p <* 0.05), suggesting the presence of visceral hyperalgesia. Treatment with EA dramatically lowered the increased AWR score in TNBS-treated mice (Figures 1A–D, *p* < 0.05). These results support the analgesic effect of EA on visceral hyperalgesia in TNBS-treated IBD mice.

In addition, compared with the vehicle control group, TNBS-treated IBD mice spent much less time in the center zone of OPF and the open arms of EPM. However, EA-treated mice spent more time in the center zone of OPF and the open arms of EPM than TNBS-treated mice (Figures 1E,F, *p* < 0.05). These results suggest that EA can reduce anxiety behaviors of TNBS-treated IBD mice. Of note, sham EA had no effects on pain and anxiety behaviors in TNBS-treated IBD mice.

### 3.2 EA Reversed Over-Expression of CB1R in GABAergic Neurons But Not Glutamatergic Neurons in the vHPC of IBD Mice

**FIGURE 2:**
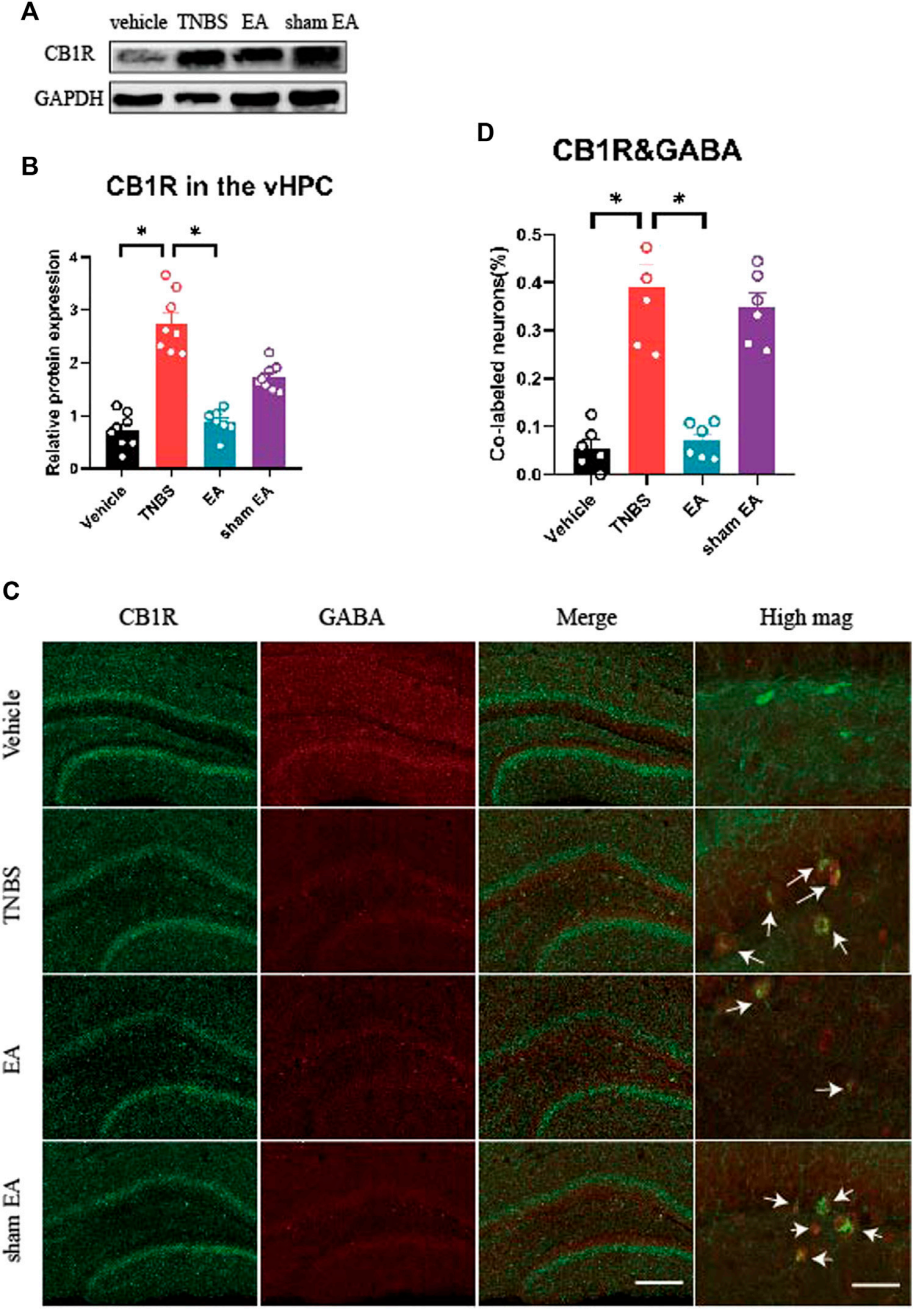
EA reversed over-expression of CB1R and decreased the percentage of CB1R-expressed GABAergic neurons in the vHPC of IBD mice. **(A)** Representative immunoblots of CB1R and GAPDH protein expression in the vHPC. **(B)** Densitometric analysis of CB1R protein normalized to the loading control. **(C)** Immunofluorescence images showed CB1R (green) co-expressed with GABA (red) in the vHPC. **(D)** Percentage of CB1R-expressed neurons co-labeled with GABA, which is (CB1R and GABA co-labeled neurons /total GABA neurons) *100%. Scale bar for merge images, 100 μm. Scale bar for high magnification images (high mag), 20 μm. The data are expressed as mean ± SEM (*n* = 9 mice). *represents *p* < 0.05 between marked groups.

The CB1R is associated with chronic pain and associated emotion disorders, such as anxiety (Davis, 2014; Patel et al., 2017; Yin et al., 2019). However, it is not clear whether CB1R in the vHPC play a role in the inhibitory effects of EA on anxiety and visceral hyperalgesia. To explore this question, we compared the expression of CB1R in the vHPC of TNBS-treated mice and vehicle control mice. Our results showed that the protein level of CB1R in the vHPC of the TNBS group was higher than that of the vehicle control group. EA reduced the protein level of CB1R in TNBS-treated IBD mice (Figures 2A,B, *p* < 0.05). These data suggest that EA reversed CB1R upregulation. However, EA have no effect on the protein level of CB1R in the amygdala (Supplementary Figures S1A,B).

**FIGURE 3:**
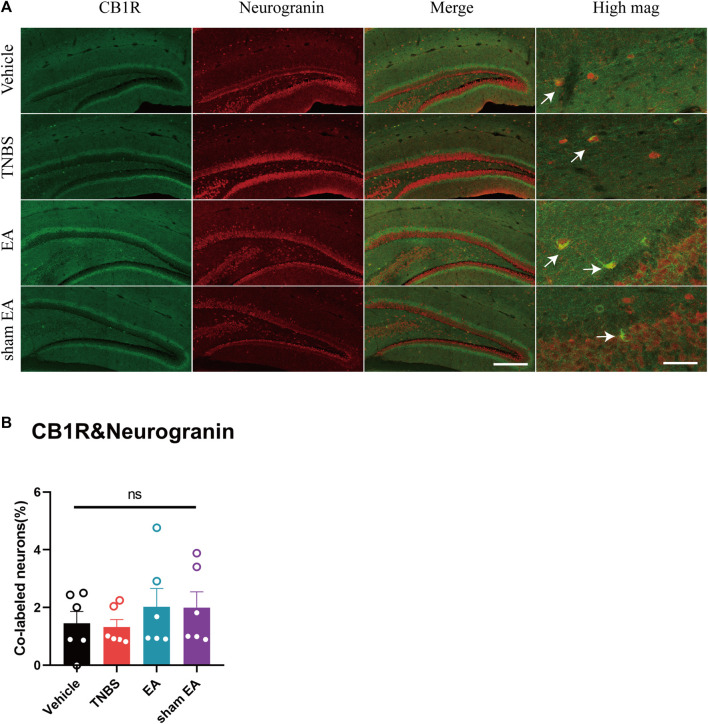
TNBS and EA had no effect on the percentage of CB1R-expressed glutamatergic neurons in the vHPC of IBD mice. **(A)** Immunofluorescence images showed CB1R (green) co-expressed with neurogranin (a marker of glutamatergic neurons, red) in the vHPC. **(B)** Percentage of CB1R-expressed neurons co-labeled with neurogranin, which is (CB1R and neurogranin co-labeled neurons /total neurogranin neurons) *100%. Scale bar for merge images, 100 μm. Scale bar for high magnification images (high mag), 20 μm. Ns represents *p* > 0.05 between marked groups.

To dissect the roles of CB1R in GABAergic or glutamatergic neurons in the vHPC, we investigated the co-localization of CB1R and GABA or neurogranin (a marker of glutamatergic neurons) (Xiang et al., 2020). Our results showed that the percentage of CB1R-expressed GABAergic neurons in the vHPC of TNBS-treated group was significantly higher than that of vehicle control group. Compared with TNBS-treated IBD mice, EA significantly reduced the percentage of CB1R and GABA co-labeled neurons (Figures 2C,D, *p* < 0.05). However, there were no difference in the percentage of CB1R-expressed glutamatergic neurons in the vHPC among four groups (Figures 3A,B).

### 3.3 Ablating CB1R in GABAergic Neurons in the vHPC Alleviated Anxiety in TNBS-Treated Mice and Mimicked the Anxiolytic Effect of EA

**FIGURE 4:**
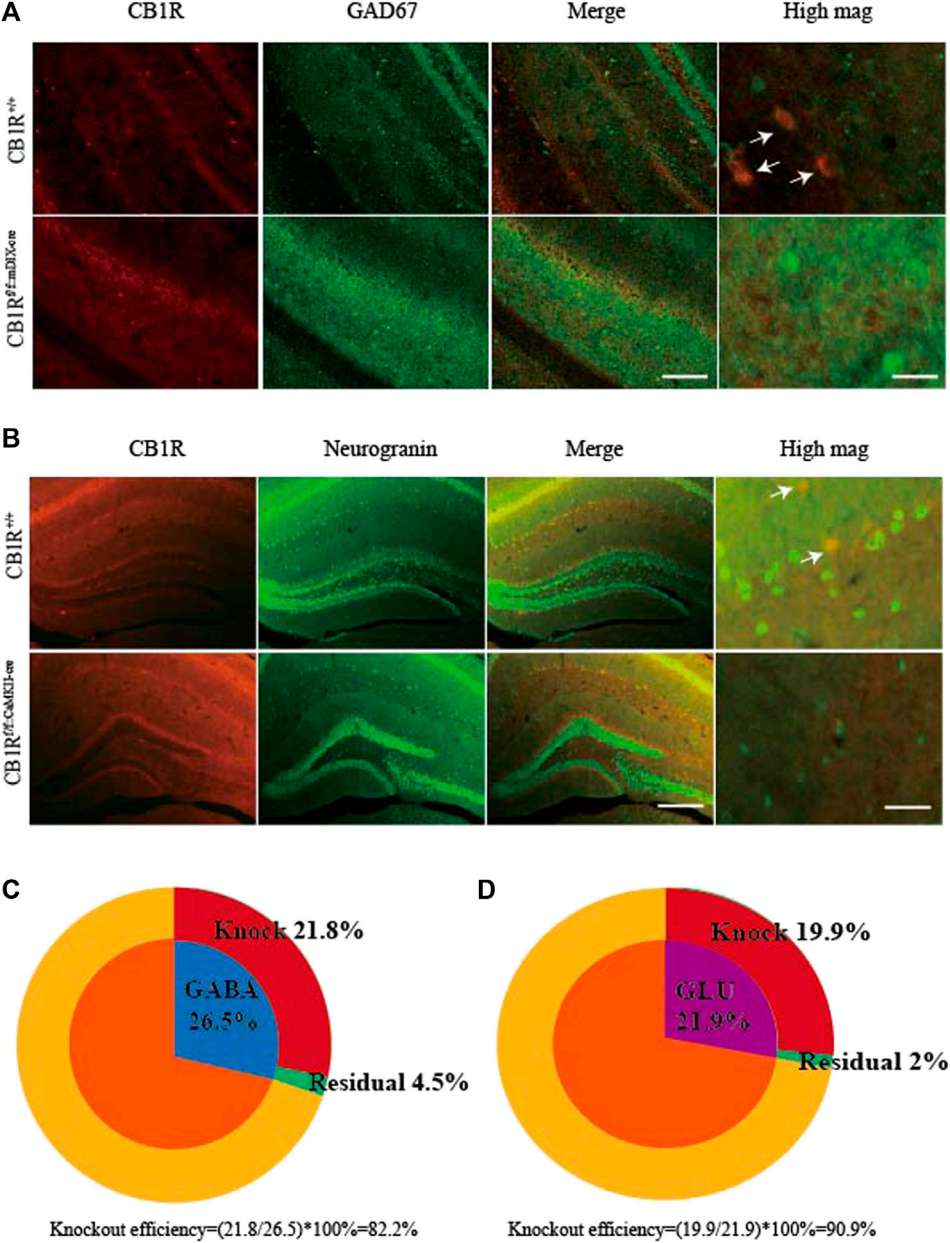
Specific knockout of the CB1R in GABAergic and glutamatergic neurons in the vHPC. **(A)** After rAAV-mDIX-cre-WPRE-pA viruses were injected into bilateral vHPC of CB1R-flox mice, immunofluorescence images showed CB1R (red) co-expressed with GAD67 (labeled GABAergic neurons, green) in the vHPC. Scale bar for merge images, 200 μm. Scale bar for high magnification images (high mag), 40 μm. **(B)** After rAAV-CaMKII-cre-WPRE-pA viruses were injected into bilateral vHPC of CB1R-flox mice, immunofluorescence images showed CB1R (red) co-expressed with neurogranin (a marker of glutamatergic neurons, green) in the vHPC. Scale bar for merge images, 100 μm. Scale bar for high magnification images (high mag), 20 μm. **(C)** Summary data show the percentage of GABAergic neurons whose CB1 receptors were knocked out (red). **(D)** Summary data show the percentage of glutamatergic neurons whose CB1 receptors were knocked out (red). Data are expressed as the means ± SEM (*n* = 3 mice in each group).

**FIGURE 5:**
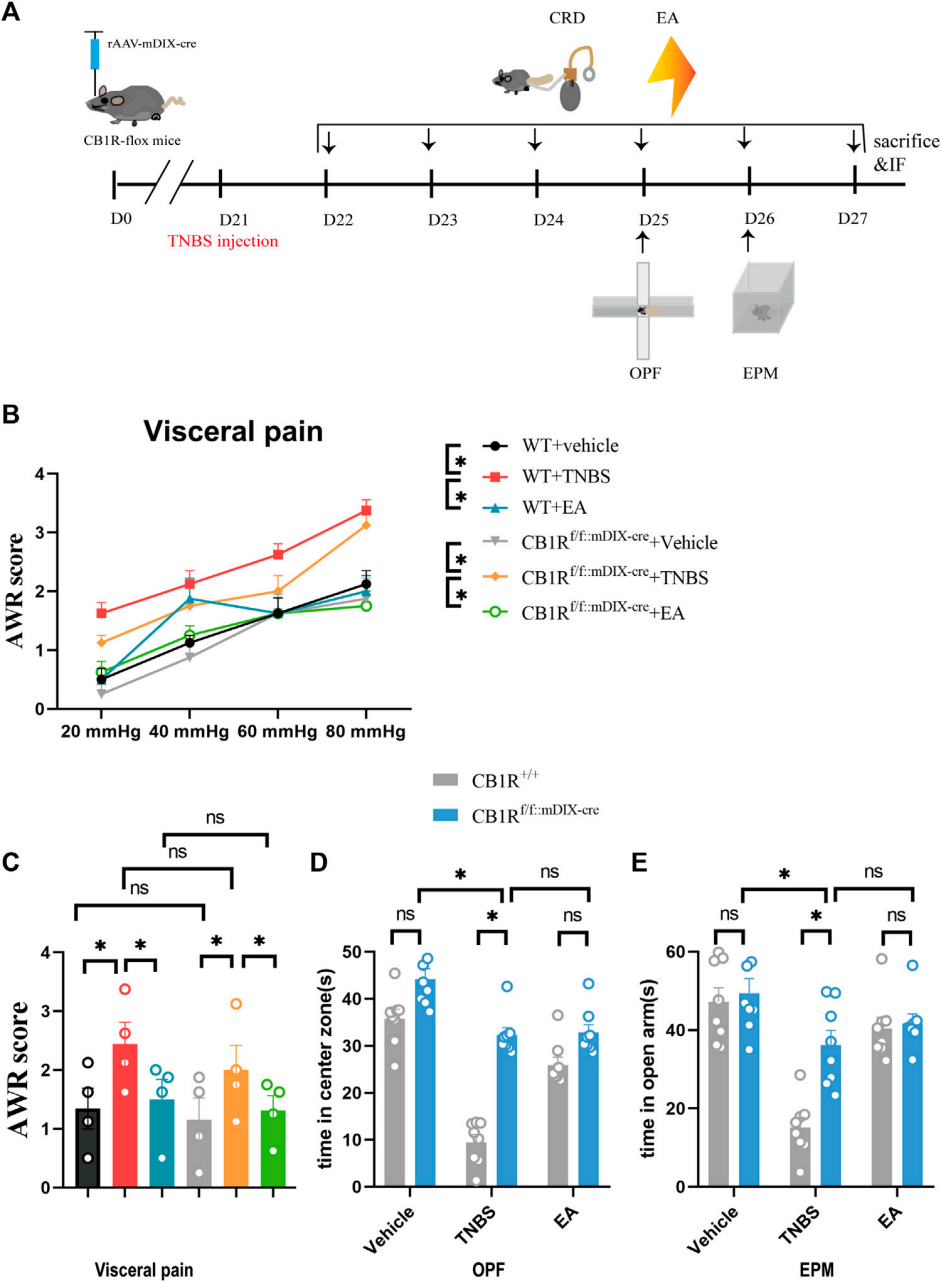
Specific knock out of the CB1R in GABAergic neurons in the vHPC mimicked the anxiolytic but not analgesic effect of EA on IBD mice. **(A)** Experimental flowchart. **(B,C)** Visceral hyperalgesia was evaluated by CRD. **(D)** Anxiety-related behaviors were recorded as time in center zone in the OPF. **(E)** Anxiety-related behaviors were recorded as time in open arms in the EPM. The data are expressed as mean ± SEM (*n* = 8 mice). * represents *p* < 0.05 between marked groups, ns represents *p* > 0.05 between marked groups.

Based on the above findings, we further determined whether EA may reduce visceral pain and anxiety associated with IBD by acting on CB1R in GABAergic neurons in the vHPC. The rAAV-mDlX-CRE-WPRE-pA viruses were bilaterally injected into the vHPC of CB1R-flox mice to ablate CB1R in GABAergic neurons in the vHPC (Figure 5A). Four weeks after viruses injection, the percentage of CB1R and GAD67 co-labeled neurons in the vHPC was decreased in the CB1R-flox mice compared with wild type mice (Figure 4A) and the knockout efficiency was 82.2% (Figure 4C). Ablating CB1R in GABAergic neurons in the vHPC had no effect on the AWR score and can’t reverse the effect of EA on visceral hyperalgesia (Figures 5A–C).

In the OPF and EPM test, CB1R deletion in GABAergic neurons in the vHPC increased the time of mice staying in the center zone and open arms in the TNBS group, compared with TNBS-treated wild type mice (Figures 5D,E, *p <* 0.05). However, there was no difference in the time of mice stayed in center zone and open arms between the TNBS group and the EA group after ablating CB1R in GABAergic neurons in the vHPC (Figures 5D,E). It is possible that the anxiolytic effect of ablating CB1R in GABAergic neurons in the TNBS group had reached a peak and could not be further increased by EA. Thus, inhibiting the expression of CB1R in GABAergic neurons in the vHPC likely mediates the anxiolytic effect of EA.

### 3.4 CB1R in Glutamatergic Neurons in the vHPC Participated in the Anxiolytic Effect of EA

**FIGURE 6:**
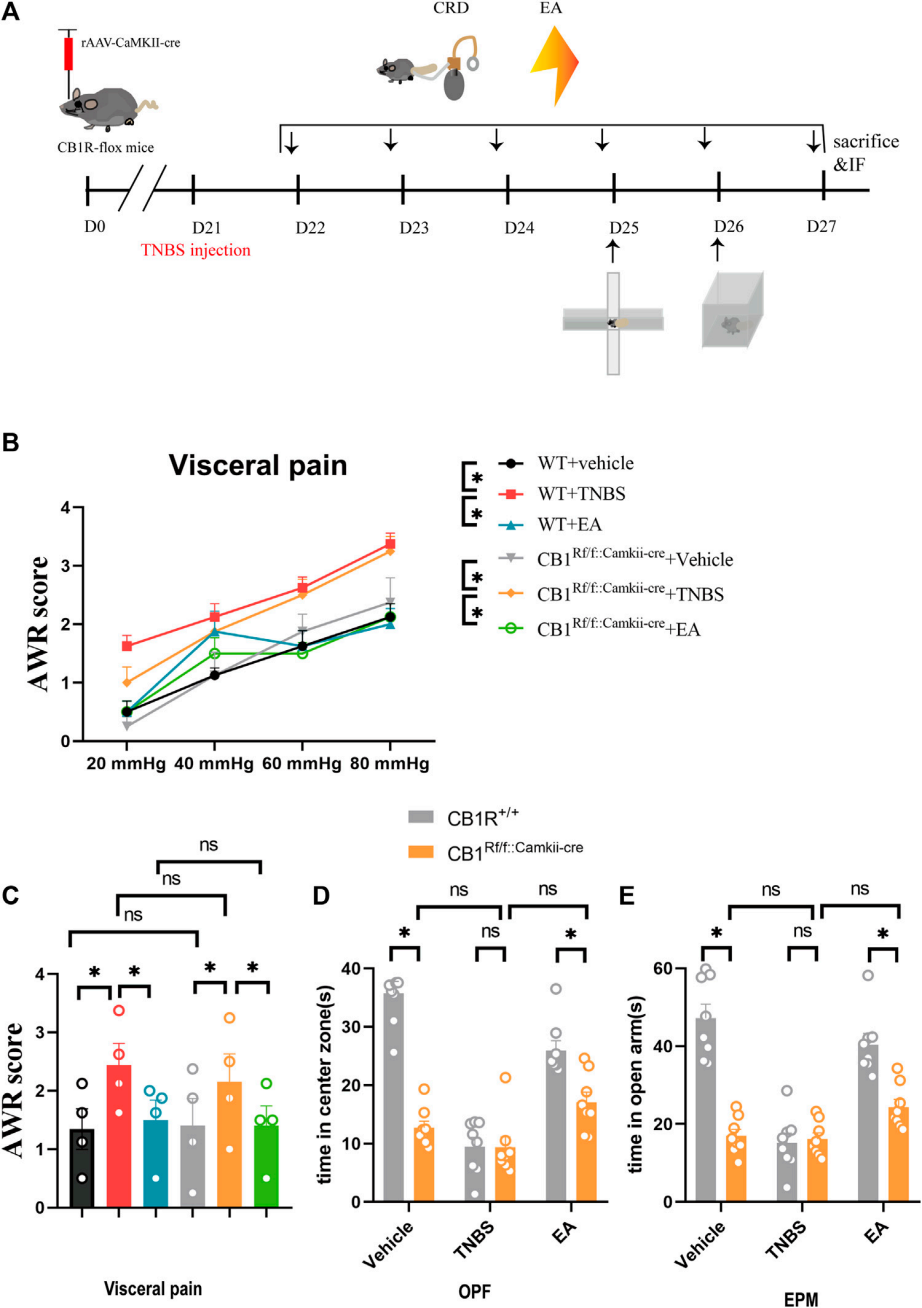
Specific knock out of the CB1R in glutamatergic neurons in the vHPC reversed the anxiolytic but not analgesic effect of EA on IBD mice. **(A)** Experimental flowchart. **(B,C)** Visceral hyperalgesia was evaluated by CRD. **(D)** Anxiety-related behaviors were recorded as time in center zone in the OPF. **(E)** Anxiety-related behaviors were recorded as time in open arms in the EPM. The data are expressed as mean ± SEM (n = 8 mice). *represents *p* < 0.05 between marked groups, ns represents *p* > 0.05 between marked groups.

In order to determine the role of CB1R in glutamatergic neurons in the analgesic and anxiolytic effects of EA, the rAAV-CaMKII-CRE-WPRE-pA viruses were bilaterally injected into the vHPC of CB1R-flox mice to ablate CB1R in glutamatergic neurons in the vHPC (Figure 6A). The percentage of CB1R and neurogranin (a marker of glutamatergic neurons) co-labeled neurons was decreased in CB1Rf/f:CaMKII-cre mice compared with wild type mice (Figure 4B) and the knockout efficiency was 90.9% (Figure 4D). Ablating CB1R in glutamatergic neurons in the vHPC did not affect AWR score and can’t reverse the effect of EA on visceral hyperalgesia (Figures 6A–C).

In the OPF and EPM tests, the time of mice stayed in the center zone and open arms was decreased in the vehicle control group but not TNBS group in CB1Rf/f:CaMKII-cre mice, compared with the corresponding groups in wild type mice (Figures 6D,E, *p* < 0.05). Moreover, after CB1R deletion in glutamatergic neurons in the vHPC, there was no difference between the vehicle control group and the TNBS group (Figures 6D,E). It is possible that vehicle control mice with CB1R deletion in glutamatergic neurons showed a high level of anxiety, which could not be further increased by TNBS treatment. The time of mice stayed in center zone and open arms in the EA group was not significantly different with the TNBS group of CB1Rf/f:CaMKII-cre mice, but was significantly lower than the EA group of wild type mice (Figures 6D,E, *p* < 0.05). These data suggested that EA attenuates anxiety *via* activation of CB1R in glutamatergic neurons in the vHPC.

## 4 Discussion

Patients with IBD have several chronic visceral disorders, including abdominal pain, rectal bleeding, and diarrhea (Gracie et al., 2018). In addition, these patients often have mood disorders, such as anxiety or depression (Walker et al., 2008; Neuendorf et al., 2016; Gracie et al., 2018). Our study found that TNBS-treated IBD mice displayed visceral hyperalgesia and anxiety-like behaviors. Several reports showed that EA relieved mechanical allodynia and visceral hyperalgesia associated with IBD (Ji et al., 2013; Lv et al., 2019; Wang et al., 2019). In addition, EA had an anxiolytic effect (Errington-Evans, 2012; Yue et al., 2018). Our present study showed that EA is effective in reducing visceral hyperalgesia and anxiety in a mouse model of IBD.

The hippocampus is not only a key region for memory and learning but also is closely involved in chronic pain and anxiety (Jones and Gebhart, 1986; Covey et al., 2000; Li et al., 2017; Parfitt et al., 2017). Direct manipulation of the hippocampus alters nociceptive behaviors (Lathe, 2001; Reckziegel et al., 2021). Moreover, hippocampus, especially vHPC has been viewed a target to treat anxiety (Li et al., 2017; Parfitt et al., 2017). The CB1R play a role in the regulation of mood disorder and chronic pain processes (Jones and Gebhart, 1986; Davis, 2014; Seltzman et al., 2016; Yin et al., 2019). In this study, EA reduced the protein level of CB1R in the vHPC of TNBS-treated IBD mice. We wonder whether CB1R in the vHPC may be involved in analgesic or anxiolytic effect of EA.

Peripherally restricted CB1R agonists may hold promise as a viable treatment for visceral pain (Di Sabatino et al., 2011). Also, CB1R in the hippocampus may be targeted for treating anxiety disorders (Jiang et al., 2005; Lisboa et al., 2015). In the hippocampus, the CB1R is present on both GABAergic and glutamatergic axon terminals (Marsicano and Lutz, 1999). Deletion of CB1R in GABAergic neurons decreases hippocampal long-term potential (LTP). In contrast, ablating CB1R in glutamatergic neurons seems to enhance hippocampal LTP (Monory et al., 2015). In order to distinguish the different effects of CB1R of excitatory and inhibitory neurons on EA, we observed the distribution of CB1R and the influence of conditional deletion of CB1R in analgesic and anxiolytic effects of EA. We found that EA reversed the upregulation of CB1R in GABAergic neurons but not glutamatergic neurons in the vHPC.

**FIGURE 7:**
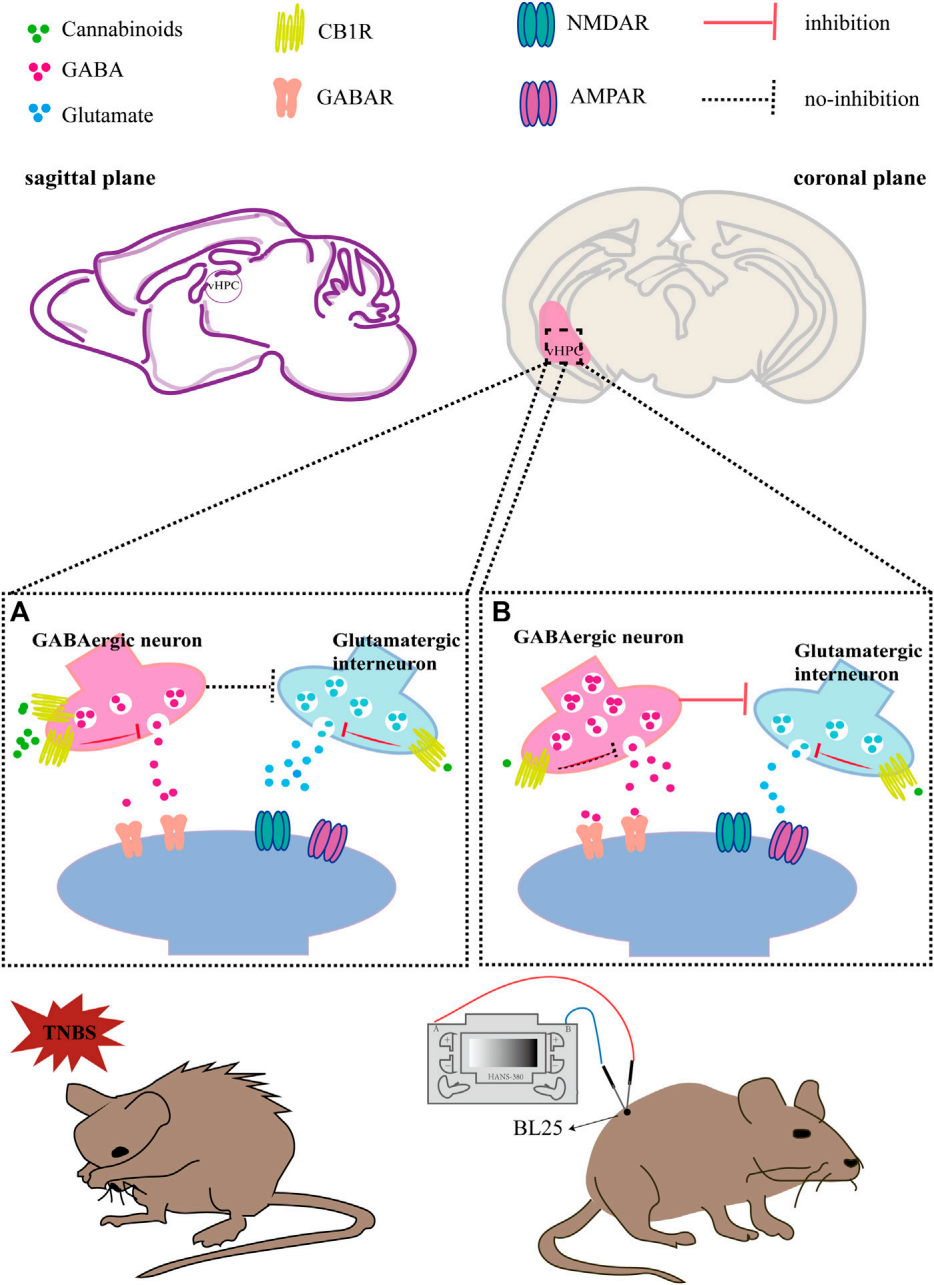
Hypothesis diagram of EA inhibiting IBD induced anxiety *via* CB1R in the vHPC. **(A)** In TNBS-induced IBD mice, CB1R was over-expressed in GABAergic neurons in the vHPC, which was activated by endocannabinoid and the release amount of GABA was reduced. As a result, the release amount of glutamate is increased because of decreased inhibition of GABA. The increased release of glutamate in the vHPC may excite anxiety-related neuronal circuits starting from vHPC, thus inducing anxiety-related behaviors in IBD mice. **(B)** In TNBS-induced IBD mice with EA treatment, EA downregulated CB1R in GABAergic neurons in the vHPC, which disinhibit the release of GABA. In turn, excessive GABA inhibited the release of glutamate. Meanwhile, CB1R in glutamatergic neurons can also be activated by EA induced endocannabinoid, which can also decrease the release amount of glutamate. To sum up, EA may exert anxiolytic effect *via* downregulating CB1R in GABAergic neurons and activating CB1R in glutamatergic neurons in the vHPC, thus reducing the release of glutamate and inhibiting the anxiety-related neuronal circuits.

In addition, our study showed that ablating CB1R of GABAergic neurons in the vHPC alleviated anxiety in TNBS-treated IBD mice and also mimicked the anxiolytic effect of EA. Previous study found that stimulating CB1R in GABAergic neurons in the vHPC can lead to an anxiogenic response *via* a decreasing GABAergic transmission (Roohbakhsh et al., 2009). We hypothesized that in TNBS-induced IBD mice, CB1R was over-expressed in GABAergic neurons in the vHPC, which was activated by endocannabinoid and reduced the release amount of GABA. Since glutamatergic neurons in the brain are usually regulated by inhibitory GABAergic neurons, the release amount of glutamate is increased because of decreased inhibition of GABA (Katona and Freund, 2008). The increased release of glutamate in the vHPC may excite anxiogenic neuronal circuits starting from vHPC, to basolateral amygdala (BLA) (Allsop et al., 2014) or the medial prefrontal cortex (mPFC) (Adhikari, 2014), thus inducing anxiety-related behaviors in IBD mice (Figure 7A). Interestingly, EA may exert anxiolytic effect by downregulating CB1R in GABAergic neurons in the vHPC, which in turn increased release of GABA and subsequently inhibited the release of glutamate, thus alleviating anxiety-like behaviors (Figure 7B).

In contrast, ablating CB1R in glutamatergic neurons in the vHPC only induced severe anxiety in vehicle control mice, but did not deteriorate anxiogenic response of TNBS-treated IBD mice. Since the CB1R in glutamatergic terminals may reduce the release amount of glutamate (Marsicano and Lutz, 1999), the reason of anxiety in vehicle control mice may be that the absence of CB1R in glutamatergic neurons in the vHPC induces excessive glutamate release, thus exciting the neuronal circuits for anxiety. Since the level of anxiety in vehicle control mice has reached a peak, it would not further increase after TNBS injection (Figure 7A). Moreover, CB1R deletion in glutamatergic neurons also inhibited the anxiolytic effect of EA. It suggested that EA may exert anxiolytic effect *via* activation of CB1R in glutamatergic neurons in the vHPC, thus reducing the release of glutamate and inhibiting the activation of neuronal circuits of anxiety (Figure 7B).

To our surprise, ablating CB1R in either GABAergic or glutamatergic neurons in the vHPC did not alter visceral hyperalgesia. It suggested that CB1R in the vHPC may not contribute to visceral pain. In our previous study, we found that EA can increase the level of endocannabinoid 2-arachidonoylglycerol in the midbrain in chronic pain, which can bidirectionally regulate GABAergic and glutamatergic neurons *via* the CB1R in the vlPAG to produce analgesic effects (Yuan et al., 2018; Zhu et al., 2019). In our study, CB1R in other brain region, such as vlPAG, may be responsible for the analgesic effect of EA in IBD induced visceral pain.

In conclusion, our findings reveal that CB1R expressed on GABAergic and glutamatergic neurons are involved in the inhibitory effect of EA on anxiety in IBD mice. EA may exert anxiolytic effect *via* downregulating CB1R in GABAergic neurons and activating CB1R in glutamatergic neurons in the vHPC, thus reducing the release of glutamate and inhibiting the anxiogenic neuronal circuits related to vHPC. Thus, our study provides new information about the cellular and molecular mechanisms of the therapeutic effect of EA on anxiety induced by IBD.

## Data Availability

The original contributions presented in the study are included in the article/Supplementary Materials, further inquiries can be directed to the corresponding authors.
