# Supplementary material for: Electroacupuncture Reduces Anxiety Associated With Inflammatory Bowel Disease By Acting on Cannabinoid CB1 Receptors in the Ventral Hippocampus in Mice
Source: Front Pharmacol. 2022 Jul 8;13:919553. doi: 10.3389/fphar.2022.919553 (PMC9305710; doi:10.3389/fphar.2022.919553)
Supplement: Supplementary file 1 [file DataSheet3.docx]

Supplementary Material

# Supplementary Data

## 1.1 EA have no effect on the protein level of CB1R in the amygdala of IBD mice.

There was no significant difference in the protein level of CB1R in the amygdala between TNBS group and vehicle control group (**Supplementary** **Figure 1A-B**), indicating that TNBS have no influence on the protein expression of CB1R in the amygdala of IBD mice.

The protein level of CB1R in the amygdala of EA group did not change significantly compared with TNBS group (**Supplementary Figure 1A-B**), indicating that EA have no effect on the protein expression of CB1R in the amygdala of IBD mice.

# Supplementary Figures and Tables

## Supplementary Figures


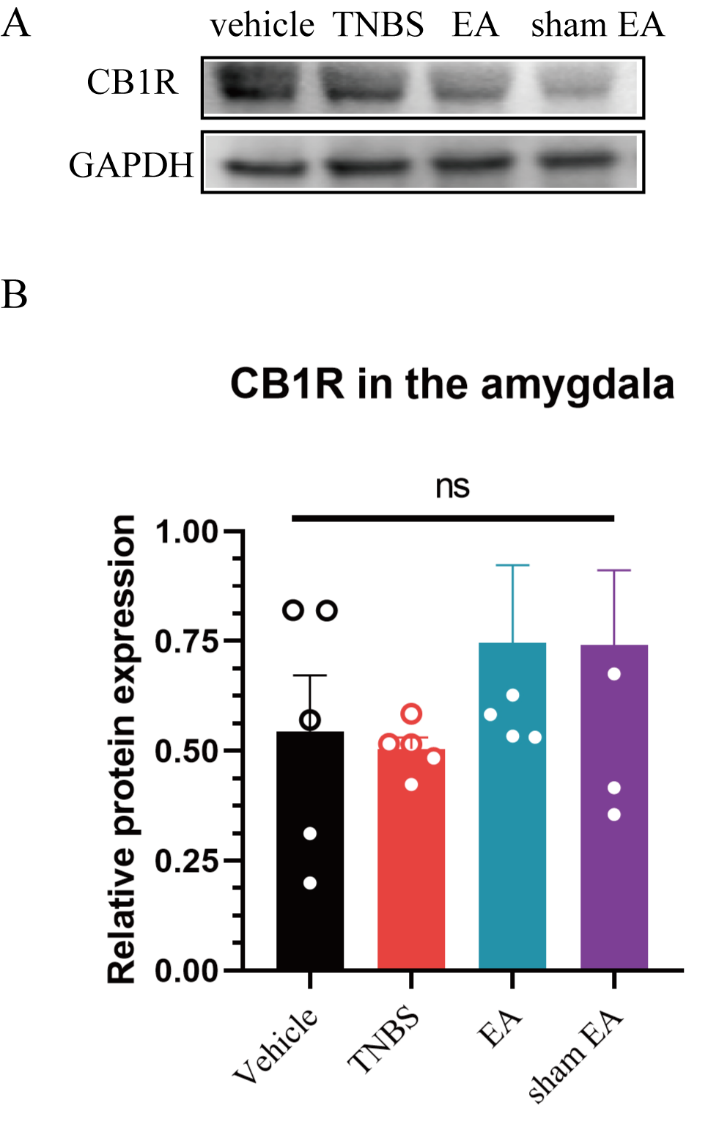


**Supplementary Figure 1.** **EA have no effect on the protein level of CB1R in the amygdala of IBD mice.** (A) Representative immunoblots of CB1R and GAPDH protein expression in the amygdala. (B) Densitometric analysis of CB1R protein normalized to the loading control. The data are expressed as mean ± SEM (n=5 mice). Ns represents P＞0.05 between marked groups.
